# Association of *SERPINC1* Gene Polymorphism (rs2227589) With Pulmonary Embolism Risk in a Chinese Population

**DOI:** 10.3389/fgene.2019.00844

**Published:** 2019-09-13

**Authors:** Yongjian Yue, Qing Sun, Lu Xiao, Shengguo Liu, Qijun Huang, Minlian Wang, Mei Huo, Mo Yang, Yingyun Fu

**Affiliations:** ^1^Key Laboratory of Shenzhen Respiratory Diseases, Department of Pulmonary and Critical Care Medicine, Shenzhen Institute of Respiratory Disease, The First Affiliated Hospital of Southern University of Science and Technology, The Second Clinical Medical College of Jinan University, Shenzhen People’s Hospital, Shenzhen, China; ^2^Shenzhen Key Laboratory of Reproductive Immunology for Peri-implantation, Fertility Center, Shenzhen Zhongshan Urology Hospital, Shenzhen, China; ^3^Research Centre, The Seventh affiliated Hospital of Sun Yat-sen University, Shenzhen, China; ^4^Department of Clinical Laboratory, The First Affiliated Hospital of Southern University of Science and Technology, The Second Clinical Medical College of Jinan University, Shenzhen People’s Hospital, Shenzhen, China

**Keywords:** SERPINC1, rs2227589, pooled systematic analysis, antithrombin anticoagulant activity, pulmonary embolism

## Abstract

**Background and Aims:** Genetic variants in the gene *SERPINC1* have been shown to be associated with antithrombin deficiency, which subsequently contributes to the susceptibility to venous thrombosis. However, several other studies have shown conflicting results regarding the association of *SERPINC1* gene polymorphisms (rs2227589) with the risk of thrombosis. Hence, in the present study, we conducted a case-control study to further evaluate the association between the variant rs2227589 with antithrombin deficiency in pulmonary embolism (PTE). A pooled systematic analysis was also conducted to evaluate the risk of rs2227589 in venous thromboembolism (VTE) among multiple populations.

**Methods:** This case-control study involved 101 patients and 199 healthy controls. The allele frequency of *SERPINC1* variant rs2227589 was analyzed by Sequenom assay. Antithrombin anticoagulant activity was detected using an automatic coagulation analyzer. In addition, a pooled systematic analysis on 10 cohorts consisting of 5,518 patients with VTE and 8,935 controls was performed.

**Results:** In total, 27 (26.7%) PTE subjects were diagnosed as having antithrombin deficiency. Our results showed that antithrombin plasma activity was slightly lower in T allele carriers than that in C allele carriers. However, there was no significant correlation between rs2227589 genotype and antithrombin anticoagulant activity. The recessive model showed that rs2227589 was significantly associated (p = 0.026) with an increased risk {odds ratio [OR]: 2.31, 95% confidence interval [CI] (1.09–4.89)} of Chinese PTE. The pooled systematic analysis of all case-control study and meta-analysis showed that rs2227589 polymorphism was associated with an increased risk of VTE in the additive model [OR: 1.09, 95% CI (1.01–1.18), P = 0.029] and dominant model [OR: 1.10, 95% CI (1.01–1.20), P = 0.034].

**Conclusions:** Our study demonstrated that variant rs2227589 is associated with an increased risk of PTE in a Chinese population but no correlation with antithrombin anticoagulant activity. However, pooled systematic analysis of multiple populations showed a significant association between rs2227589 and the risk of VTE in the additive and dominant genetic model.

## Introduction

Venous thromboembolism (VTE) is a complex and common cardiovascular disease that includes deep venous thrombosis (DVT), cerebral infarction, and pulmonary embolism (PTE), which is caused by multiple factors. The incidence of VTE is around 0.1–0.2% in Caucasian and American populations (Beckman et al., 2010). The hospitalization rates of VTE increased from 3.2 to 17.5 per 100,000 population, and the mortality decreased from 4.7% to 2.1% in China (Zhang et al., 2019). The acquired or inherited risk factors for the development of VTE include surgery, pregnancy, and cancer (Heit, 2015; Hotoleanu, 2017). Familial cohort and case-control studies have demonstrated that VTE is often familial or hereditary (Mili et al., 2011; Holzhauer et al., 2012). Over 60% of the variations in susceptibility to common thrombosis are attributable to genetic factors (Souto et al., 2000).

Genetic variations in coagulation system genes (such as *F5*, *PROC*, and *SERPINC1*) contribute to susceptibility to venous thrombosis (Garcia de Frutos et al., 2007; Rosendaal and Reitsma, 2009). Protein C (PROC), protein S (PROS1), and antithrombin have been demonstrated to play important roles in the anticoagulation process (Lee et al., 2017). *SERPINC1* is the gene encoding antithrombin. Deficiency of antithrombin is usually caused by rare or private variations of *SERPINC1* gene. Deficiency among coagulation system factors can increase the risk of developing thrombosis. Inherited antithrombin deficiency is a rare, autosomal dominant disorder (MIM#107300). Antithrombin exerts its physiological function by inhibiting procoagulation factors, such as thrombin, factor Xa, factor IIa, and other factors of the blood coagulation system (Zeng et al., 2015). Antithrombin belongs to the serine protease inhibitor superfamily and regulates clot formation both by inhibiting thrombin activity directly and by interfering with earlier stages of the clotting cascade (Rosenberg and Bauer, 1987). There are two types of antithrombin deficiency. In type I antithrombin deficiency, functional and antigenic levels are proportionally decreased. In type II antithrombin deficiency, antigenic levels are normal while the functional activity is abnormal. In around 0.02–0.25% of a healthy population with antithrombin deficiency, there is a 5- to 50-fold increased risk of developing venous thrombosis (Zhu et al., 2011; Kim et al., 2014; Luxembourg et al., 2014).

The first variation linked to antithrombin deficiency was characterized in 1983 and, to date, more than 200 variants have been reported to be associated with the risk of thrombosis (Corral et al., 2007; Navarro-Fernandez et al., 2016). The homozygous variant (Phe229Leu) of *SERPINC1* leading to spontaneous antithrombin polymerization *in vivo* has been shown to be associated with severe childhood thrombosis (Picard et al., 2003). The heterozygous variant is mainly associated with a high risk of venous thrombosis (Arruda et al., 1997; Navarro-Fernandez et al., 2016). However, most of these variants are rare and seldom replicate in other populations. rs2227589, a polymorphism of *SERPINC1* gene (NG_012462.1:g.5301G > A), was found to be associated with the risk of venous thrombosis in a Dutch population (Bezemer et al., 2008). The minor allele frequencies of rs2227589 is 0.10 (gnomAD) and 0.329 in the East Asian population. Previous study of normal Spanish Caucasian showed a functional effect of the rs2227589 on antithrombin levels (Anton et al., 2009). The findings regarding the association between rs2227589 and antithrombin levels were inconsistent among different studies (Segers et al., 2014; Bhakuni et al., 2015). Furthermore, the association between rs2227589 and the risk of VTE in Northern European, American, and Norwegian populations is inconsistent (Tregouet et al., 2009; Austin et al., 2011; Dahm et al., 2012; Jiang et al., 2017; Kajuna et al., 2018). The study of Jiang did not enroll a large sample size as in the study of Tregouet et al. (2009) and showed negative results with publication bias (Jiang et al., 2017). Similarly, another Swedish population study showed no significant association between rs2227589 and the risk of VTE (Bruzelius et al., 2015). These findings imply that associations might differ between Western and Asian populations depending on ethnicity.

Given that the association between rs2227589 and the Chinese PTE population has not been examined, we performed this case-control study to investigate the association between variant rs2227589, antithrombin deficiency, and PTE risk in a Chinese population. Furthermore, a systematic review and a meta-analysis were carried out to evaluate the variant in antithrombin deficiency and VTE.

## Materials and Methods

### Study Subjects

A total of 98 patients with PTE, 3 patients with familial VTE, and 199 matched controls were recruited from the Second Clinical Medical College of Shenzhen People’s Hospital from December 2013 to September 2017. The three familial VTE subjects were enrolled because each had more than two siblings who were diagnosed with PTE. Strict criterion was conducted to exclude the multiple PTE effected risk factors in this association analysis, such as cancer, diabetes, and so on. In this regard, only 101 cases were enrolled in our studies. The inclusion criteria for the PTE patients were the criteria released by the European Society of Cardiology (ESC) in 2014 (Konstantinides, 2014). Most of the recruited subjects are unprovoked, recurrent, or inherited acute PTE. The healthy controls were recruited as described previously (Yue et al., 2019). Informed consent was obtained from each patient. Research and ethics approval was obtained from the ethics committee of Shenzhen People’s Hospital. All procedures involving human participants were performed in accordance with the 1964 Declaration of Helsinki. Demographic characteristics and medical histories were recorded, including age and history of family disease.

### Plasma Antithrombin Anticoagulant Activity Assay in PTE Subjects

Blood samples were collected into trisodium citrate by venopuncture. Plasma samples were obtained by centrifugation at 2,000g for 15 min at 4°C and stored at -80°C until analysis. The antithrombin anticoagulant activity was determined by automatic chromogenic assay on the IL Coagulation systems according to the manufacturer’s instructions (Cat.0020300400, Werfen, Bedford, MA). Briefly, the plasma was first incubated with Factor Xa reagent in the presence of an excess of heparin, followed by incubation with a synthetic chromogenic substrate. Quantification of the residual Factor Xa was performed by detecting the absorbance at 405 nm by ACLTOP 700 system (Werfen). The absorbance is inversely proportional to the antithrombin level in the test sample. The activation levels were calculated by the standard curve of each detection. The established normal range of plasma antithrombin levels was 83–128%, which was based on our hospital pathology reference from thousands of previously diagnosed clinical subjects.

### DNA Preparation and Genotyping

Genomic DNA was extracted from whole blood using QIAamp DNA Blood kit (Qiagen, Hilden, Germany) according to the manufacturer’s instructions. The candidate single-nucleotide polymorphism (SNP) was genotyped by MassARRAY SNP genotyping platform (Sequenom, San Diego, CA) among PTE case and control subjects. The following primers for polymerase chain reaction (PCR) amplification were used: forward, 5′-ACGTTGGATGGAAAGGCCTTACCCCAAGAG-3′ and reverse, 5′-ACGTTGGATGTCTCCCTGGTAGTTACAGTC-3′. The genotyping assay extension primers for SNP rs2227589 were 5′-GGAGAGCACTTGAAATGAT-3′. All primers were designed by Sequenom’s MassARRAY Designer software. A specific primer extension was performed to detect single-base polymorphisms in the amplified DNA.

### Systematic Review Analysis

A systematic literature search was performed using PubMed, Science Direct, ISI Web of Knowledge, Google Scholar, and the CNKI Database, and the cutoff date was defined as January 2019. A meta-analysis was performed in accordance with the Preferred Reporting Items for Systematic Reviews and Meta-Analyses (PRISMA) (Moher et al., 2009). Search terms using the Boolean operators at PubMed included but were not limited to the following: “venous thrombosis” (MeSH Terms) OR VTE (All Fields) AND *SERPINC1* (MeSH Terms) OR rs2227589 (All Fields). Only case-control studies regarding an association between rs2227589 and VTE were considered eligible. The data extracted from the eligible studies included author, publication year, racial and genotype methods, and variants frequency. The control population genotype data of publications followed the Hardy-Weinberg equilibrium.

### Eligibility Criteria and Data Validity Assessment

The eligibility criteria of studies are according to our previous publication (Yue et al., 2019). The criterion for including eligible studies was that the DVT or PTE should be diagnosed with standard criteria in the case group. Only case-control studies regarding an association between rs2227589 and VTE were considered eligible. For exclusion, reviews, conference abstracts, republished or duplicate studies, and meta-analyses were not included. The data extracted from the eligible studies included author, publication year, racial and genotype methods, and variants frequency. The control population genotype data of publications followed the Hardy-Weinberg equilibrium. The validity of data was evaluated by three independent reviews. The genotype results of two eligible studies were not complete or available in the publications. Finally, the genotype data were kindly shared by professor Pierre-Emmanuel Morange (Tregouet et al., 2009), but the other study results were not obtained from the authors upon request (Heit et al., 2017).

### Statistical Analysis and Meta-Analysis

All statistical analyses were performed using software STATA 14.0 and SPSS 19 (IBM). The strength of association was evaluated by pooled odds ratio (OR) and 95% confidence interval (CI). Either a fixed-effects model (Mantel-Haenszel method) or a random-effects model was used for meta-analysis. The *z*-test was used to evaluate the significance of the pooled OR (Lewis, 2002). Heterogeneity was tested using Cochran’s Q statistic (Higgins et al., 2003), and publication bias was tested using Egger’s test (Egger et al., 1997; Sterne et al., 2001). Nonparametric trim-and-fill method was performed to assess the possible effect of funnel plot asymmetry and publication bias in the meta-analysis (Duval and Tweedie, 2000). Sensitivity analysis was conducted by sequentially omitting one study at a time. Chi square and Bonferroni (*post hoc*) tests were used for cross-tabulation and multiple comparisons, respectively. P < 0.05 was considered statistically significant.

## Results

### Demographics of the Participants

After filtering with the inclusion criteria and exclusion criteria, a total of 101 patients were enrolled for plasma antithrombin anticoagulant activity level assay in this study. The general characteristics of PTE patients are presented in **Table 1**. The mean age of the patients with PTE was 59 ± 1.7 years old. Antithrombin activity deficiency was present in 27 patients (26.7%) with venous thrombosis. The average level of active antithrombin was 90 ± 2.0%. The details of antithrombin anticoagulant activity in patients with PTE were presented in **Supplemental Table S1**.

**Table 1:** Demographics of the participants and antithrombin anticoagulant activity in patients with pulmonary embolism.

Characteristic/Genotype	Number (%) or mean ± SEM	Antithrombin activity (Mean ± SEM)	*p* value
Gender Male/female(n)	51/50		
Age (years)	59 ± 1.7		
Total subjects	101	90 ± 2.0%	83–128% (normal range)
Deficiency case	27	62.9 ± 2.8%	P < 0.001
Normal case	84	99.9 ± 1.4%	
rs2227589 genotype			
CC	40 (39.6%)	91.6 ± 2.8%	P > 0.05
CT	45 (44.6%)	88.5 ± 3.5%	
TT	16 (15.8%)	90.3 ± 4.6%	

### Association of Genetic Models Between rs2227589 and PTE Risk

**Figure 1:**
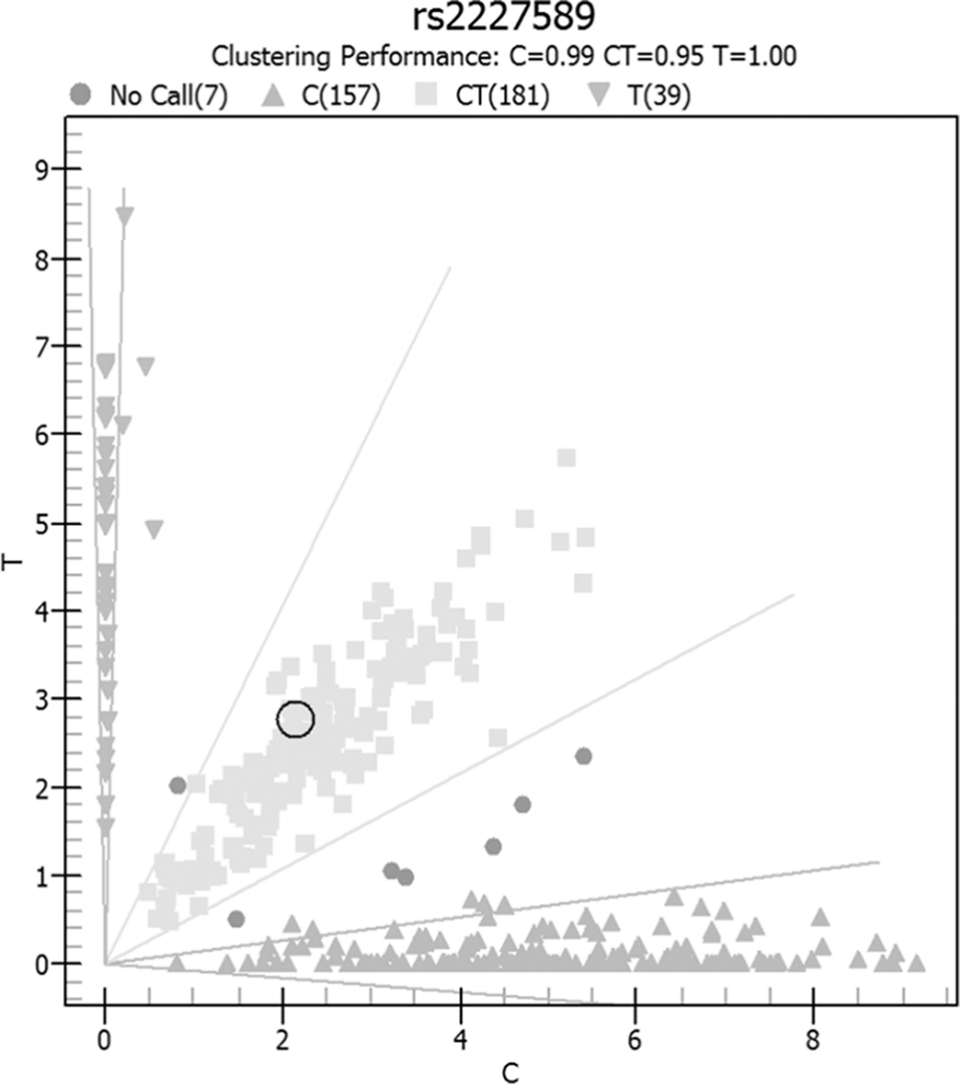
Genotype distribution of rs2227589 in pulmonary embolism and normal subjects.

Allele and genotype frequencies of *SERPINC1* polymorphism (rs2227589) in PTE patients and controls are summarized in **Figure 1** and **Table 2**. The genotypes of rs2227589 followed the Hardy-Weinberg equilibrium among the controls. In our study, the CT genotype (heterozygote) was 44.6%, which was much higher than in the previously reported ethnic cohorts (around 20%). Furthermore, homozygote SNPs were also present in the PTE group at a very high frequency (>7%). The frequencies of the SNP homozygote and heterozygote were similar between the case and control groups. Carriers of *SERPINC1* polymorphism (rs2227589) were associated (P = 0.026) with an increased risk of PTE [OR: 2.31, 95% CI (1.09–4.89)], which was detected in the recessive model but not in the additive and dominant models (**Table 3**).

**Table 2:** Genotype distribution of *SERPINC1* polymorphism (rs2227589) among different studies.

Author	Years	Ethnicity	Source	Case			Control			Methods
				CC	CT	TT	CC	CT	TT	
Bezemer	2008	Netherlands	Mega2	1,001	278	15	2325	483	28	PCR
Tregouet	2009	Northern European	GWAS	317	89	5	983	235	10	Beadchip
Tregouet	2009	Northern European	MARTHA	922	228	12	621	149	12	Taqman
Tregouet	2009	Northern European	FARIVE	478	111	5	467	119	1	Taqman
Austin	2011	White America	White	426	113	5	530	127	7	Taqman
Austin	2011	Black America	Black	469	58	2	499	71	5	Taqman
El-Galaly, TC	2013	Danish	White	494	105	8	1,434	279	17	Taqman
Rovite, V	2014	Latvia	Latvia	139	38	0	179	53	3	Taqman
Kajuna, I	2018	Latvia	Latvia	82	17	0	71	27	1	Taqman
Yue, YY	2019	Asian	Chinese	40	45	16	84	100	15	Sequenom

**Table 3:** OR, 95% CIs, and antithrombin activity levels in three genetic models of rs2227589 among patients with pulmonary embolism.

Genetic models		Pooled effect		Antithrombin activity	
Models	Allele	OR (95% CI)	P	Mean ± SEM	P
Additive	T vs. C	1.27 (0.89–1.81)	0.18	88.9 ± 2.9 vs. 89.9 ± 2.3	0.79
Dominant	TT + TC vs. CC	1.11 (0.68–1.81)	0.67	89.0 ± 2.9 vs. 91.6 ± 2.8	0.25
Recessive	TT vs. TC + CC	2.31 (1.09–4.89)	0.026	86.8 ± 3.7 vs. 90.6 ± 2.3	0.5

### Association Assay Between Plasma Antithrombin Anticoagulant Activity and Frequency of rs2227589 Carriers

The correlation between rs2227589 and antithrombin anticoagulant activity of PTE was also evaluated. Among rs2227589 carriers, 16 (16/61) showed antithrombin deficiency compared with noncarriers (11/40) (**Table 1**). Only 4 subjects showed antithrombin deficiency among the 16 homozygote SNP carriers. There was no significant difference in antithrombin activity levels among the three rs2227589 genotype groups. Carriers of the TT alleles had slightly lower anticoagulant activity than did carriers of the CC alleles, but this was not statistically significant (P = 0.45). We also checked whether significant differences existed among the associated recessive genetic model groups (CC + CT vs. TT). The results showed that the T allele carriers had slightly lower antithrombin activity, but this was not statistically significant (**Table 3**).

### Meta-Analysis for the Association Between rs2227589 and VTE Risk in Different Populations

**Figure 2:**
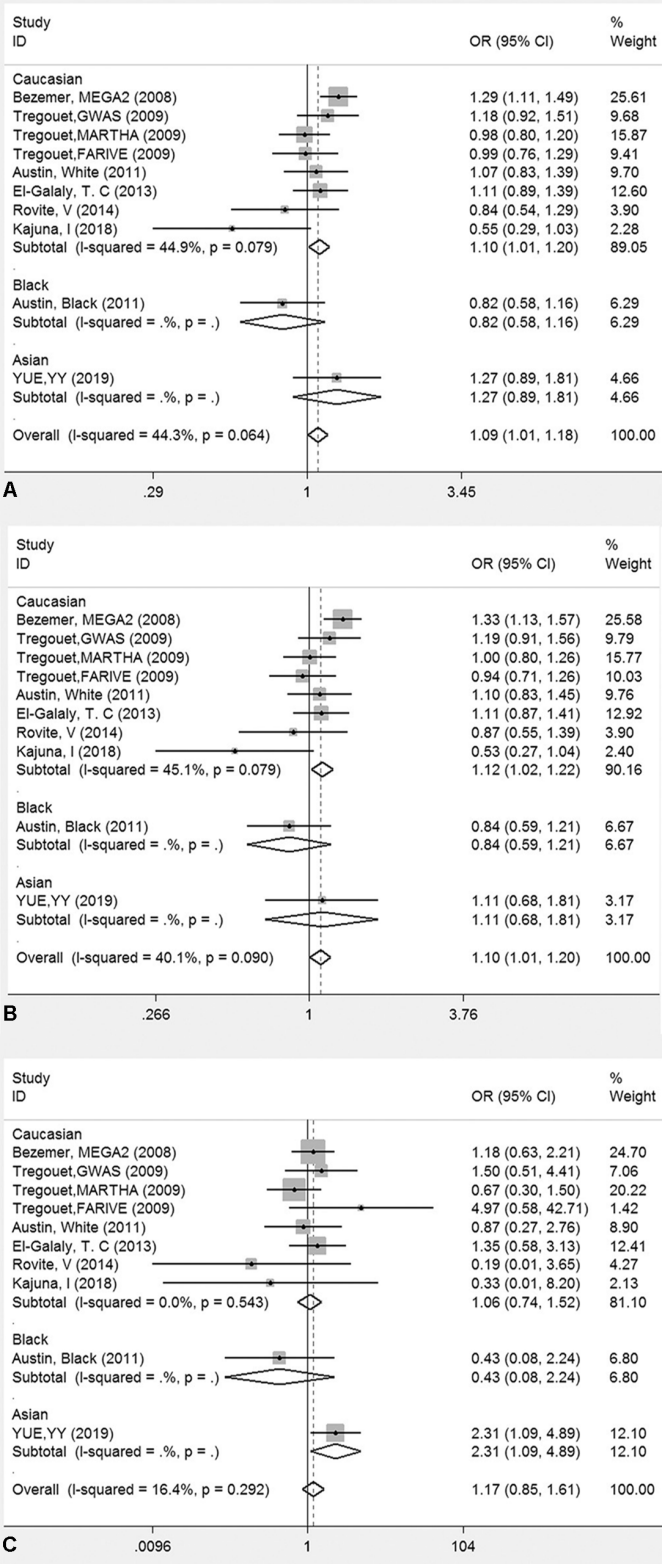
Forest plots for the association between rs2227589 and the risk of venous thromboembolism among different populations **(A)**, additive model; **(B)** dominant model; **(C)** recessive model).

**Figure 3:**
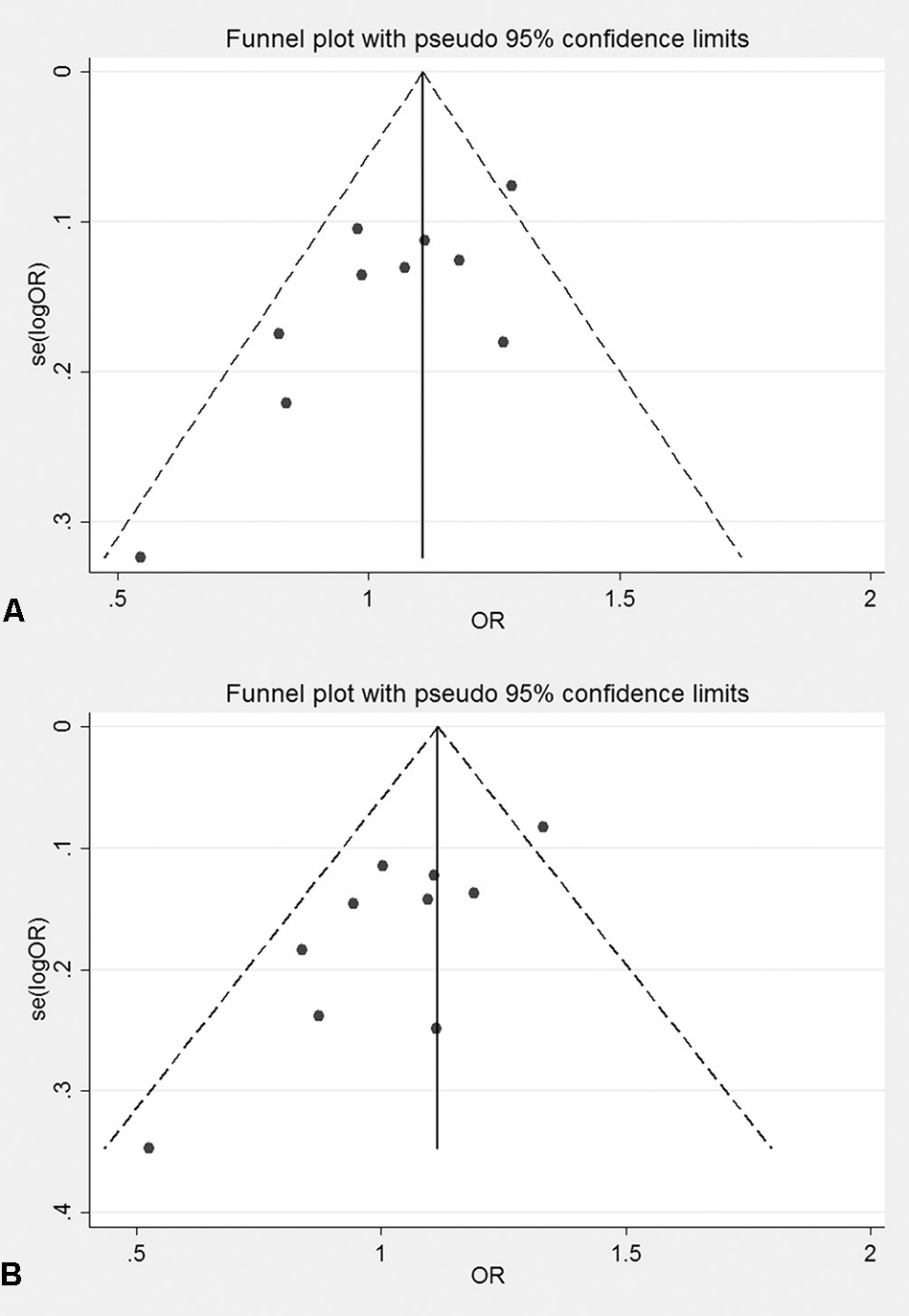
Funnel plot of bias examination in the additive model **(A)** and dominant model **(B)**.

**Figure 4:**
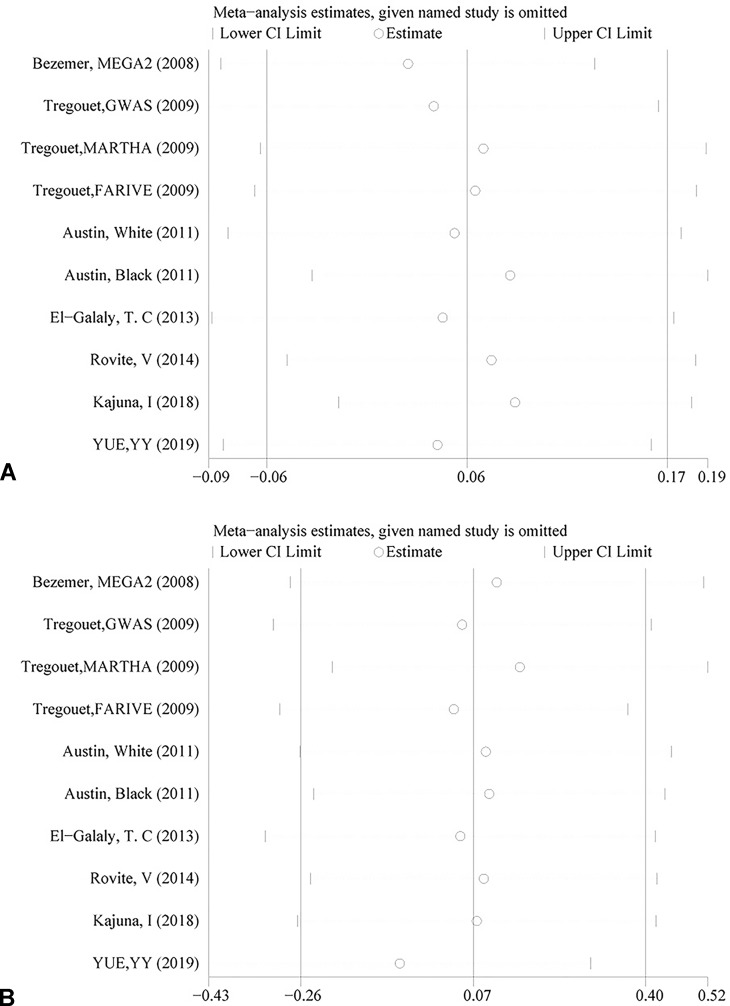
Sensitivity analysis of rs2227589 and the risk of venous thromboembolism (VTE) in the additive model **(A)** and dominant model **(B)**.

The genotyping data of rs2227589 in 101 Chinese PTE patients of our case-control study were enrolled in the meta-analysis. In pooled systematic analysis, 10 case-control cohorts from six studies were enrolled. The total numbers of VTE-affected patients and controls were 5,518 and 8,935, respectively. The characteristics, genotype distribution, and allelic frequencies of the eligible studies are displayed in **Table 2**. The meta-analysis was carried out using the additive, recessive, and dominant genetic models. The heterogeneity value was less than 50% (P > 0.05), indicating that our study had low heterogeneity and that a fixed model could be used in the analysis (**Table 4**). The association effect distribution is presented as forest plots (**Figure 2**, **Supplementary Figure S1**). The association effect of pooled OR with the additive model was 1.09 (95% CI 1.01–1.18, P = 0.029) and the dominant model was 1.10 (95% CI 1.01–1.20, P = 0.034). Systematic analysis of previous cohort studies showed a significant association between rs2227589 and the risk of VTE under additive and dominant genetic models. Subgroups analysis of Caucasians showed consistent results of the association (**Table 4**). Furthermore, potential publication bias was examined by funnel and Galbraith plots (**Figure 3**; **Supplemental Figure S2**). The results showed that there was no sharp asymmetry between the two models, which indicated no obvious potential bias among the enrolled publications. But Galbraith assay showed potential bias of additive and dominant models, which indicated that the bias may be caused by the small sample size or ethnic differences of enrolled cohorts. Thus, trim-and-fill method analysis was applied to correct publication bias. The results showed the pooled effect unchanged of the two genetic models and continued to be statistically significant, which indicated that the effect of bias is very slight with reliable conclusions (**Supplemental Figure S3**). Sensitivity analyses showed that the pooled ORs fluctuated among confidence intervals (**Figure 4**), which suggested good reliability among our results and methods to provide an effective evaluation.

**Table 4:** The results of pooled OR, 95% CIs, and heterogeneity by meta-analysis.

Genetic models		Pooled effect		z	Heterogeneity	
Models	Allele	OR (95% CI)	P_z_		I^2^ (%)	P_H_
Additive	T vs. C	1.09 (1.01–1.18)	0.029	2.18	44.3	0.06
	Subgroups (Caucasian)	1.10 (1.01–1.20)	0.023	2.27	44.9	0.08
Dominant	TT + TC vs. CC	1.10 (1.01–1.20)	0.034	2.21	40.1	0.09
	Subgroups (Caucasian)	1.12 (1.02–1.22)	0.017	2.39	45.1	0.08
Recessive	TT vs. TC + CC	1.17 (0.85–1.61)	0.328	0.98	16.4	0.29
	Subgroups (Caucasian)	1.06 (0.74–1.52)	0.741	0.33	0.0	0.54

## Discussion

Because of controversial results from GWAS and case-control studies of rs2227589, a prediction of the risk factors associated with developing VTE is unreliable. In this study, we explored the association between the variant rs2227589 with antithrombin deficiency and risk of PTE in a Chinese population. Our case-control study found a significant association between *SERPINC1* rs2227589 polymorphism and increased risk of PTE in the recessive model. Pooled systematic analysis of all cohorts showed a significant association in the additive and dominant genetic models. The association between rs2227589 and repeated episodes of VTE demonstrated a genetic risk with ethnic differences.

Inherited antithrombin deficiency is an autosomal dominant thrombotic disorder associated with potential risk factors for the development of DVT. Antithrombin is a plasma serine protease inhibitor that can progressively inactivate thrombin, FIIa, and FXa anticoagulation functions. A functional study of rare variations in *PROC*, *PROS1*, and *SERPINC1* genes or other coagulation factors suggests that rare variants may cause inherited deficiencies in the anticoagulant system. Previous studies in an Asian population reported that the incidence of antithrombin deficiency in VTE was around 5.9%–9.61% (Liu et al., 1994; Zheng et al., 2009). Most VTE subjects with antithrombin deficiency have been found to carry genetic variants of *SERPINC1* (Zeng et al., 2015; Mulder et al., 2017). However, the functional mechanism of variant rs2227589 underlying the levels of antithrombin in plasma remains largely unknown (Anton et al., 2009).

Our studies showed that the incidence of antithrombin deficiency is 26.7% in PTE. In our study, the definition of antithrombin deficiency was based on our established antithrombin deficiency normal range (83–128%). The time point of the sample collection, the definition of antithrombin deficiency, and the different selection of patients are the several factors that may affect the frequency of antithrombin deficiency (Bucciarelli et al., 2012; Di Minno et al., 2014). The high prevalence of antithrombin deficiency in our study may be caused by these factors. The minor allele frequencies of the variant rs2227589 is 0.329 (gnomAD) in the East Asian population but not reported in the Chinese VTE population before. Our genotype data showed that the frequency of rs2227589 was also two times higher than that found in other studies. However, correlation analysis and comparison study of plasma antithrombin activity levels and genotype showed no significant association. The limitation of our study was the relatively small sample size because of the limited sources for us to recruit more patients. More patients recruited from multicenters may consolidate our findings and draw a more reliable conclusion. A study in a Spanish Caucasian population showed that healthy carriers of the rs2227589 SNP T allele had slightly but significantly lower anticoagulant activity with low antithrombin levels (Anton et al., 2009). Moreover, rs2227589 with haplotype in the *SERPINC1* gene of *FV Leiden* carriers showed significantly decreased antithrombin levels (Segers et al., 2014). However, another familial study showed that both rs2227589 carriers and noncarriers have low antithrombin levels (Bhakuni et al., 2015). Thus, variations located in the coding regions of *SERPINC1* but not the rs2227589 (C > T) located in the intron may cause antithrombin deficiency. Thus, whether there is an association between rs2227589 and antithrombin levels requires further investigation. The functional consequences of *SERPINC1* rs2227589 polymorphism might be directly caused by the regulatory effects of this genetic change or caused by other genetic variants linked to the rs2227589 polymorphism. Nevertheless, our study showed consistency with previous studies in that T allele carriers of VTE had slightly lower antithrombin levels, although this was not significant.

Our meta-analysis showed a significant association between rs2227589 and the risk of VTE in additive and dominant genetic models. Indeed, in our initial recruitment, we have recruited 225 patients. Our study showed that the genotyping distributions of rs2227589 in Chinese VTE and PTE groups are similar. When we included all of the 225 patients for meta-analysis, we found that the outcomes of meta-analysis (see **Supplemental Tables 2**, **3** and figures for the reanalyzed data) were consistent with the ones using 101 patients for analysis, suggesting the reliability of our findings. Genome-wide and familial case association studies have shown that numerous common or rare variants contribute to the risk of VTE (Smith et al., 2009). For common variants, a GWAS study already identified polymorphisms associated with the risk of DVT, such as *CYP4V2* (rs13146272), *SERPINC1* (rs2227589), and *GP6* (rs1613662) (Bezemer et al., 2008; Tregouet et al., 2009). Our study showed that carriers with low frequent TT genotype had a much higher risk of VTE based on the recessive model. The first GWAS reported by Bezemer et al. showed that carriers with the low-frequency T allele have a modest thrombotic risk in LETS, MEGA-1, and MEGA-2 case-control studies (Bezemer et al., 2008). This finding was later confirmed in the study by Austin et al. (2011). However, other studies failed to find any association between rs2227589 (*SERPINC1*) and VTE (Tregouet et al., 2009; Dahm et al., 2012; El-Galaly et al., 2013; Bruzelius et al., 2015; Heit et al., 2017). Thus, whether rs2227589 plays any role in the risk of VTE remains controversial.

In conclusion, our study showed that variant rs2227589 is associated with an increased risk of PTE in a Chinese population. However, there appears to be no correlation with antithrombin deficiency in PTE. Further validation by employing a larger sample size and multiple-center studies may provide a more reliable conclusion of the association. Pooled systematic analysis showed a significant association between rs2227589 and the risk of VTE in the additive and dominant genetic models of multiple populations. As the contribution of rs2227589 to the risk of VTE may vary with different PTE ethnicities, further investigations into the genetic risk factors and phenotype interactions would be needed to improve the prognosis, prevention, and genetic counseling for populations at high risk of VTE.

## Ethics Statement

The project was approved by the ethics committee of the Ethics Committee of Shenzhen People’s Hospital. All procedures performed in studies involving human participants were in accordance with the 1964 Declaration of Helsinki ethical standards. Written informed consent was obtained from the patients for the publication of the patient’s identifiable information.

## Author Contributions

YY and YF prepared the project proposal and study design. YY and QS analyzed all of the genotyping data and conducted the statistical analysis. LX and SL conducted sample collection. QH, MW, and MH conducted antithrombin activity detection experiment. MY assisted with the preparation and revision of the manuscript. All of the authors have read and approved the final manuscript.

## Funding

The study was supported by the National Natural Science Foundation of China (21807072), the Guangdong Provincial Natural Science Foundation (2018A030310674), the Shenzhen Science and Technology Project (JCYJ20170413093032806, JSGG20170414104216477), and the Guangdong Provincial Science and Technology Project (2017A020214016).

## Conflict of Interest Statement

The authors declare that the research was conducted in the absence of any commercial or financial relationships that could be construed as a potential conflict of interest.
