# Supplementary figures and images for: Association of SERPINC1 Gene Polymorphism (rs2227589) With Pulmonary Embolism Risk in a Chinese Population
Source: Front Genet. 2019 Sep 13;10:844. doi: 10.3389/fgene.2019.00844 (PMC6753222; doi:10.3389/fgene.2019.00844)

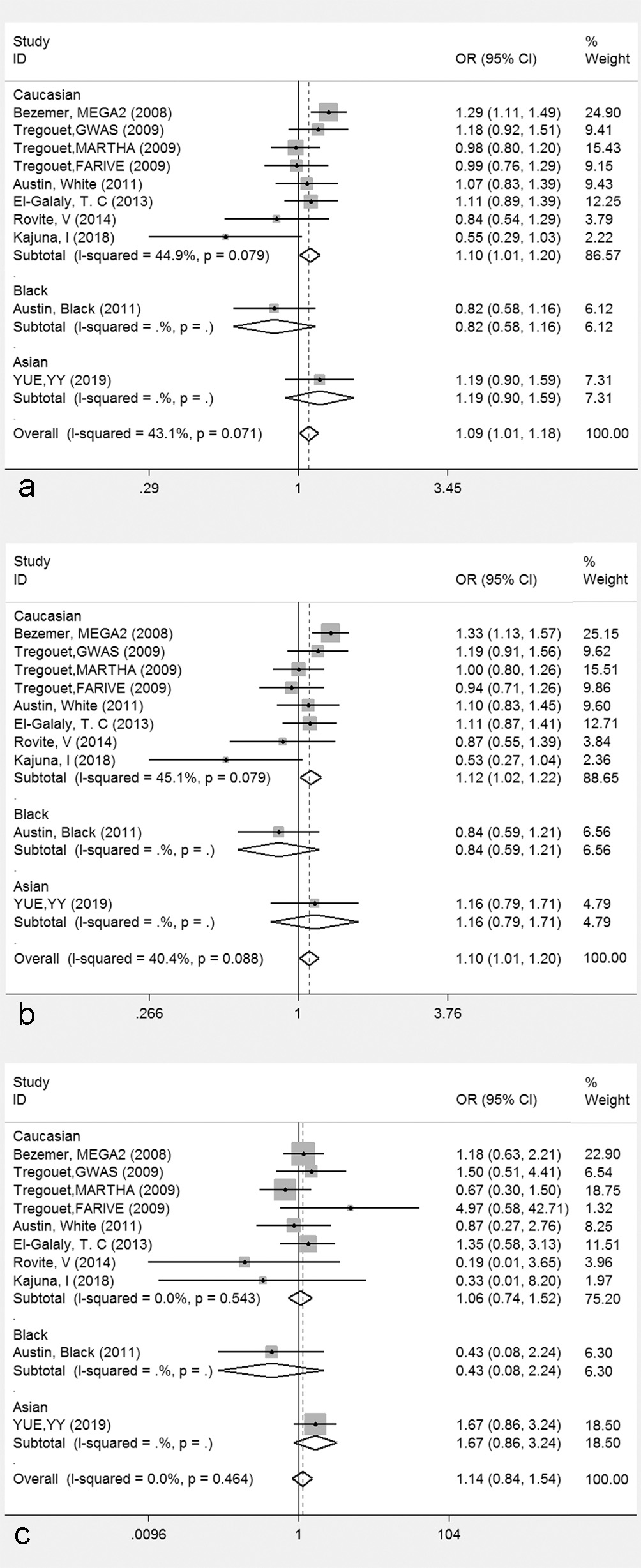

Supplement: Supplementary file 1 [file Image_1.jpeg]

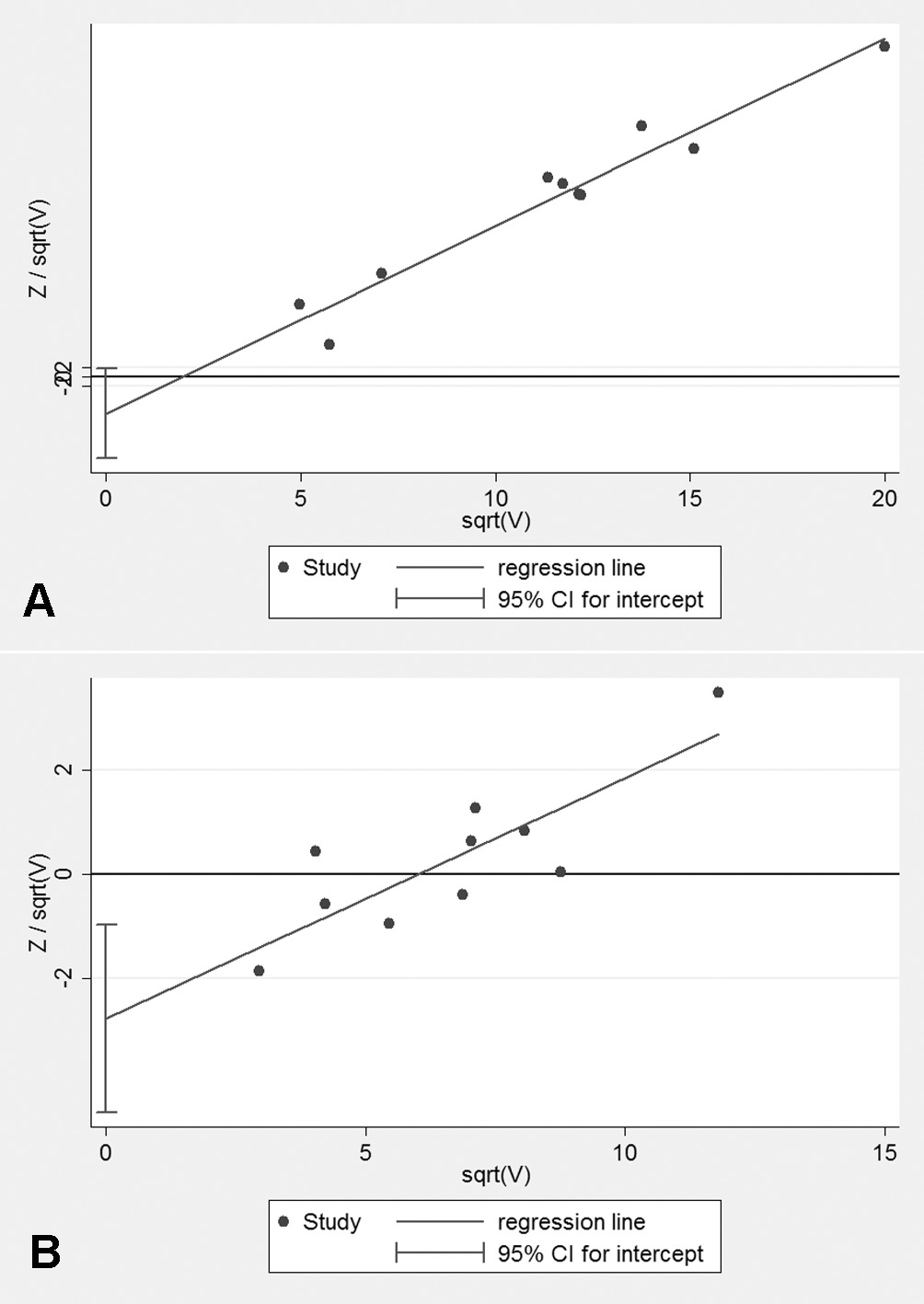

Supplement: Supplementary file 2 [file Image_2.jpeg]

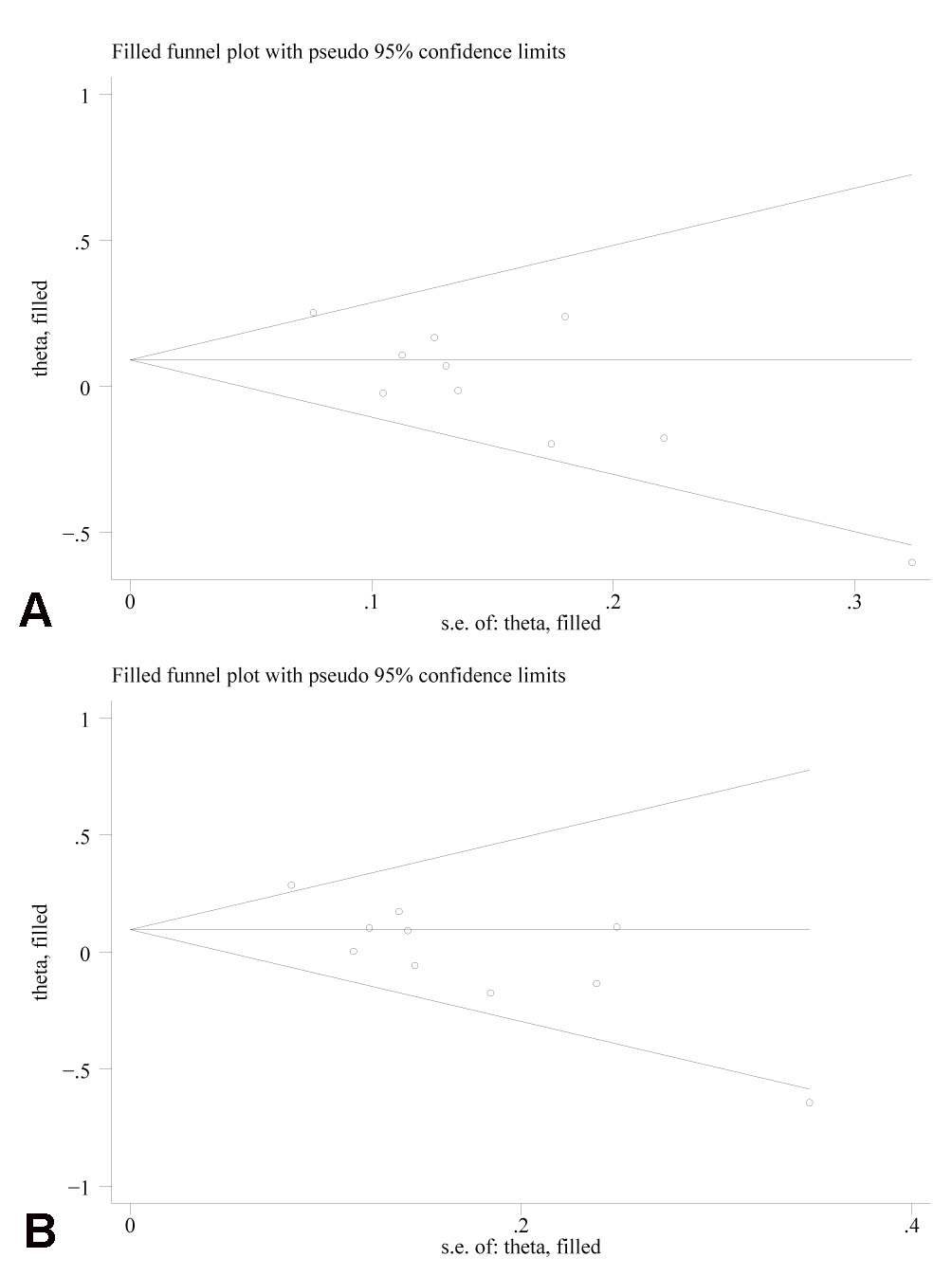

Supplement: Supplementary file 3 [file Image_3.jpeg]
