# Supplementary material for: Association of SERPINC1 Gene Polymorphism (rs2227589) With Pulmonary Embolism Risk in a Chinese Population
Source: Front Genet. 2019 Sep 13;10:844. doi: 10.3389/fgene.2019.00844 (PMC6753222; doi:10.3389/fgene.2019.00844)
Supplement: Supplementary file 5 [file Table_2.docx]

Table S2. Genotype results of *SERPINC1* polymorphism (rs2227589) among all recruited Chinese VTE subjects

| Author | Years | Ethnicity | Source | Case | | | Control | | | Methods |
| --- | --- | --- | --- | --- | --- | --- | --- | --- | --- | --- |
|  |  |  |  | CC | CT | TT | CC | CT | TT |  |
| YUE,YY | 2019 | Asian | Chinese | 87  (38.7%) | 111  (49.3) | 27  (12%) | 84  (42.2%) | 100  (50.3%) | 15  (7.5%) | Sequenom |

Table S3. The results of pooled OR, 95% CIs and heterogeneity of all VTE subjects by meta-analysis

| Genetic models | | Pooled effect | | z | Heterogeneity | |
| --- | --- | --- | --- | --- | --- | --- |
| Models | Allele | OR(95%CIs) | P_z_ |  | I^2^(%) | P_H_ |
| Additive | T vs C | 1.09(1.01-1.18) | 0.028 | 2.20 | 43.1 | 0.07 |
|  | Subgroups (Caucasian) | 1.10(1.01-1.20) | 0.023 | 2.27 | 44.9 | 0.08 |
| Dominant | TT+TC vs CC | 1.10(1.01-1.20) | 0.028 | 2.19 | 40.4 | 0.09 |
|  | Subgroups (Caucasian) | 1.12(1.02-1.22) | 0.017 | 2.39 | 45.1 | 0.08 |
| Recessive | TT vs TC+CC | 1.14(0.84-1.54) | 0.416 | 0.81 | 0.0 | 0.46 |
|  | Subgroups (Caucasian) | 1.06(0.74-1.52) | 0.741 | 0.33 | 0.0 | 0.54 |

Fig S1. Forest plots for the association between rs2227589 and risk of VTE among different populations (a, additive model; b, dominant model; c, recessive model).

Fig S2. Galbraith assay plots for the association between rs2227589 and risk of VTE (A, additive model; B, dominant model).

Fig S3. Trim-and-fill assay plots of genetic models (A, additive model; B, dominant model).
